# Expression of Cytochrome c3 from *Desulfovibrio vulgaris* in Plant Leaves Enhances Uranium Uptake and Tolerance of Tobacco

**DOI:** 10.3390/ijms222312622

**Published:** 2021-11-23

**Authors:** Denis V. Beliaev, Dmitry V. Tereshonok, Nina F. Lunkova, Ekaterina N. Baranova, Ekaterina S. Osipova, Stepan V. Lisovskii, Galina N. Raldugina, Vladimir V. Kuznetsov

**Affiliations:** 1 K. A. Timiryazev Institute of Plant Physiology RAS, 127276 Moscow, Russia; diman_ter_vi@mail.ru (D.V.T.); nina.lunkova@gmail.com (N.F.L.); oes@bk.ru (E.S.O.); vlkuzn@mail.ru (V.V.K.); 2 N.V. Tsitsin Main Botanical Garden of Russian Academy of Sciences, Botanicheskaya 4, 127276 Moscow, Russia; greenpro2007@rambler.ru; 3 All-Russia Research Institute of Agricultural Biotechnology, 127550 Moscow, Russia; 4 Moscow Institute of Physics and Technology, 141707 Moscow, Russia; lisovskii.sv@mipt.ru

**Keywords:** uranium, phytoremediation, genetic engineering, *Nicotiana tabacum*, cytochrome c3

## Abstract

Cytochrome c3 (uranyl reductase) from *Desulfovibrio vulgaris* can reduce uranium in bacterial cells and in cell-free systems. This gene was introduced in tobacco under control of the RbcS promoter, and the resulting transgenic plants accumulated uranium when grown on a uranyl ion containing medium. The uptaken uranium was detected by EM in chloroplasts. In the presence of uranyl ions in sublethal concentration, the transgenic plants grew phenotypically normal while the control plants’ development was impaired. The data on uranium oxidation state in the transgenic plants and the possible uses of uranium hyperaccumulation by plants for environmental cleanup are discussed.

## 1. Introduction

Uranium, a heavy metal, is a natural component of earth and waters, yet it has no role in living organisms and is toxic to them. Elevated uranium in the environment, whether naturally found or of anthropogenic origin, is a matter of public concern [1]. Phytoextraction, removal of contaminants from the environment using plants, is an alternative to mechanical cleanup of contaminated soils and waters, and its advantage is that the heavy metals can be concentrated in growing plants, with possibly additional concentration when the contaminated plants are dried and burnt [2]. There are many examples of soil [3] and water [4] decontamination using plants, unmodified or genetically engineered [5]. To date, however, phytoremediation of uranium was attempted, repeatedly, only with unmodified plants (see [6] for an example). Electrochemical reduction of heavy metals is viewed as an approach to bioremediation. Metals in less positive oxidation states are less toxic [7] and may be easier to recycle. Genetic engineering of plants to reduce and thus diminish the toxicity of heavy metals has had some successes. For instance, Rugh et at. [8] expressed a bacterial mercuric reductase *merA* in poplar, and the transgenic plants developed tolerance to ionic mercury. When grown on mercury- supplemented media, *merA*-plants released metallic mercury at much higher rates compared to controls. Uranium, however, is an especially difficult metal to bioremediate. Uranium uptake by plants is greatly dependent on either secreted [9] or externally added [10] organic anions, citrate or lactate. Specific pathways of uranium uptake and translocation by plants remain to be elucidated. By all means, uranium in oxidation state +6 takes form of uranyl ion UO_2_^2+^, which is water soluble and can migrate, whereas uranium in oxidation state +4 takes form of an insoluble mineral uraninite UO_2_ or other insoluble minerals and cannot migrate.

Cytochrome c3 (cytc3) from a sulfur bacterium *Desulfovibrio vulgaris* str. Hildenborough can reduce uranium U(VI) to U(IV). This enzyme works in purple bacteria [11] and in a cell-free system [12]. When it was expressed in E.coli, the *ccm* operon expression was required for the holocytochrome c3 assembly [13]. The *ccm* operon encodes enzymes for cytochrome c3 periplasmic targeting, post-translational modifications, folding and haems insertion [14].

Both classical and recent [15,16,17,18] literature data point to biochemical similarity of mitochondrial and chloroplast intermembrane spaces and bacterial periplasm. We hypothesized that cytochrome c3 from *D. vulgaris* would work in plant mitochondria provided that it is properly expressed, targeted, folded and assembled. These transgenic plants would be expected to reduce uranyl to uraninite and hyperaccumulate uranium. To test this hypothesis, cytochrome c3 was expressed in a model plant species tobacco, and uranium accumulation in the above-ground plant organs and uranium tolerance of the cytc3 transgenic plants were demonstrated as evidence of the protein function.

## 2. Results

**Figure 1:**
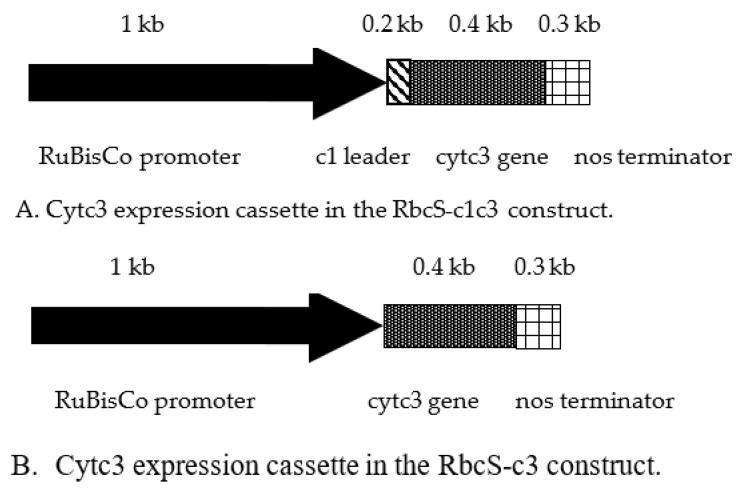
The maps of cytochrome c3 expression cassettes that were inserted in pBI121 (Clontech, Mountain View, CA, USA) in lieu of the 35S-GUS cassette.

The aim of this work was to test a hypothesis that cytochrome c3 from *D. vulgaris* can reduce uranium and thus help to hyperaccumulate this element as this is being done by *D. vulgaris*. The details of genetic constructs for expression of this cytochrome in transgenic tobacco are given in Figure 1 and in the Supplementary Materials. Several issues have been addressed for efficient expression of the functional cytochrome c3 from *D. vulgaris* in plants for uranium reduction and hyperaccumulation. For efficient translation, the gene sequence of the mature cytochrome c3 [19] was checked for the absence of codons rarely used in nuclear-coded plant mRNAs. The sequence had no codons with usage frequency less than 25% of the most frequently used codon usage frequency (data not shown). In *D. vulgaris*, cytochrome c3 is targeted to periplasm with its N-terminal peptide cleaved off [20,21]. In a plant cell, intermembrane spaces of mitochondria and chloroplasts are analogous to bacterial periplasm and have the components of cytochrome holoenzyme and the enzymes for its assembly. To target cytochrome c3 to intermembrane space (IMS) of mitochondria, two strategies were employed. The first one is to fuse it to an N-terminal leader known to target proteins to IMS. Cytochrome c reductase from potato is nuclear-encoded and targeted to IMS of mitochondria [22], and the N-terminal leader of this protein was fused in frame with cytochrome c3 for targeting to mitochondrial IMS. This chimeric protein named c1c3 was expressed in tobacco plants using a genetic construct RbcS-c1c3 (Figure 1A). Alternatively, the mature cytochrome c3 was expressed without any leader from a cassette named RbcS-c3. The outer mitochondrial membrane is permeable for proteins up to about 60k Da [23], the value well above the size of mature cytochrome c3 of 13,976 Da (Available online: https://www.uniprot.org/uniprot/P00131 (accessed on 22 November 2021)).

In *D. vulgaris*, cytochrome c3 propeptide is cleaved before an Ala residue of the mature protein [23]. To express a mature cytochrome c3, no amino acids were added at its N-terminus. Instead, a Met codon 10 amino acids downstream of the N-terminal peptide site of cleavage in *D. vulgaris* was used for translation initiation. It was reasoned that the other ways to express the mature protein from bacteria in plants without its ingenious leader would involve adding an initiation codon at the N-terminus of the mature protein or replacing some amino acid(s) with an initiation codon. Either of these three approaches may affect the folding and ultimately, function of the protein, as would expression of the protein in cytoplasm rather than by the *D. vulgaris* ribosomes at the bacterial membrane do. No motifs important for disulfide bridges characteristic of the native cytochrome c3 were found in ten amino acids preceding the Met codon used by us to express the protein from the RbcS-c3 construct [23]. In both our constructs, cytochrome c3 expression was driven by the promoter of ribuloso bisphosphate carboxylase-oxygenase gene from tobacco [24]. Tissue unspecific expression of cytochrome c3 would have resulted in uranium reduction and hyperaccumulation in roots, and this phenomena would be hard to discern from uranium bound to the root surface as was the case elsewhere [25]. Thus, there were two reasons to use the RbcS promoter that works in green tissues and not in roots [26]. The first reason was the idea that reduction of uranium from soluble uranyl to insoluble uraninite in the above-ground organs may create a gradient of uranyl in xylem with higher concentrations in roots helping to elevate uranyl up the plant. The second reason was that uranyl accumulating in leaves is highly toxic whereas its reduced form, uraninite is insoluble and thus is less toxic [1]. The construct sequences are given and annotated in Supplementary materials file.

The cytochrome c3 expressing genetic constructs were stably introduced in *Nicotiana tabacum* cv. Petit Havana by *Agrobacterium*-mediated transformation. The presence of the transgene was verified by PCR (data not shown). The primary regenerants (T0) were pot-grown and selfed repeatedly, and their T3 progeny that were all Km-resistant were used for further analyses. It was reasoned that these T3 plants should have come from T2 homozygous transformants, otherwise the Km resistance phenotype would have been segregating and some T3 plants would be Km sensitive.

**Figure 2:**
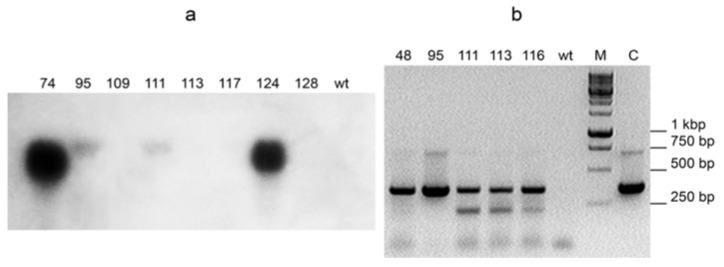
Expression of cytc3 RNA detected by Northern hybridization (**a**) or RT-PCR (**b**). RbcS-c1c3 was introduced in lines 74, 111, 48 and 116. RbcS-c3 was introduced in lines 95, 113 and 124. wt is RNA from an untransformed plant.

Expression of cytochrome c3 was analyzed in T0 transformants by Northern hybridization and/or RT-PCR. As expected, the expression levels varied greatly (Figure 2). Although Northern hybridization gives quantitative estimate of mRNA content, it is sometimes not as sensitive as RT-PCR. So, Northern hybridization of c3-116 plant RNA with the cytochrome c3 probe produced no detectable signal, but RT-PCR of the same RNA sample did (see Figure 2a,b). 

**Figure 3:**
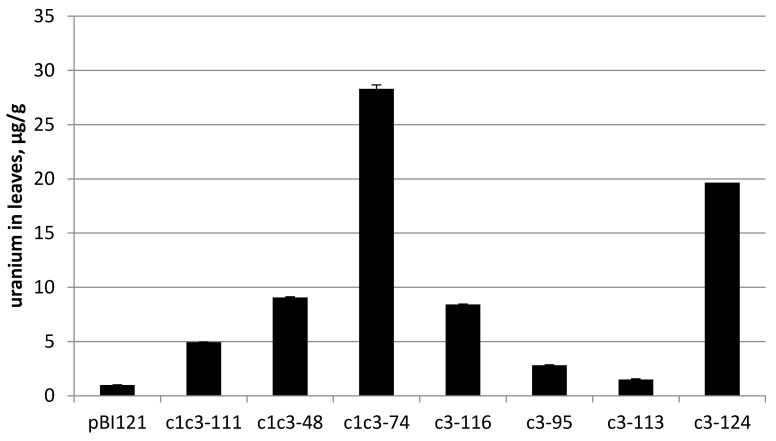
Uranium content of different transgenic lines, per fresh weight. pBI121—control line, the other lines are designated as construct name-line number. RbcS has been omitted from construct names for brevity.

The transgenic lines carrying either of RbcS-c1c3 or RbcS-c3 with confirmed cytochrome c3 expression were grown from the T3 seeds on MS medium supplemented with 0.2 mM uranyl acetate until they reached the lids of Magenta boxes. The plants weighed from 1 to 5 g at harvest. The leaves were then collected, dried and incinerated. The ashes were dissolved in 10 mL of 2% nitric acid and their uranium content was measured by ICP-MS. Regardless of the construct, all analyzed transgenic lines accumulated more uranium than the control line transformed with pBI121 construct without the target gene, but to the greatly varying extent. For instance, uranium content of the line c3-113 merely exceeded that of the control line by 50%. Uranium concentration in this plant per fresh weight was only about 2% of uranium concentration in the growth medium, but the best-performing lines c1c3-74 and c3-124 accumulated uranium to the levels comparable with its concentration in the medium (20–28 µg/g fresh weight versus 48 µg/g of medium) (Figure 3).

Note that these best lines carry different constructs, with and without the N-terminal leader, respectively. As judged by Northern hybridization and RT-PCR semi-quantitative measurements of cytc3 mRNA content, cytc3 expression levels and uranium accumulation in leaves did not always positively correlate among the analyzed transgenic lines. However, the lines with the strongest expression of cytc3, c1c3-74 and c3-124, also were superior to the other lines regarding uranium accumulation. On the other end, the line named c3-113 had cytc3 expression low enough to be undetectable by Northern, and although it accumulated more uranium than the control plants, it had the lowest uranium in leaves compared with the other transgenic lines. The observed correlation presents further evidence of cytc3 functioning in the plants. For the lines with expression measured by Northern hybridization, the expression/uranium accumulation coefficient was 0.83 (see Table S1 in Supplementary materials).

**Figure 4:**
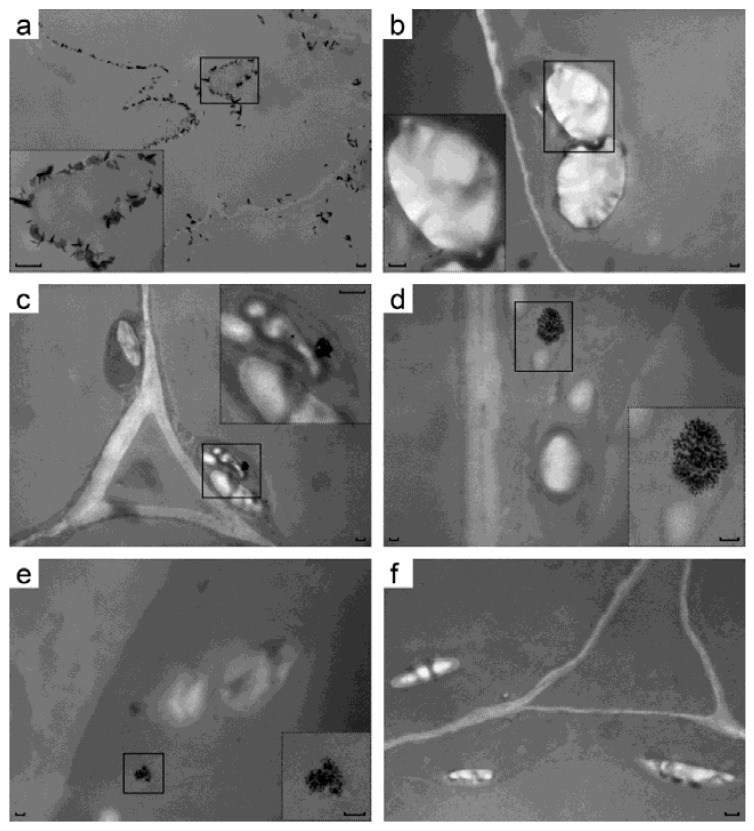
TEM micrographs of samples of transgenic tobacco plants grown on 500 µM uranyl acetate. (**a**) roots of pBI121 (control) line; (**b**) leaves of pBI121 (control) line; (**c**–**e**) leaves of the c1c3-48 line; (**f**) leaves of the c3-116 line. Scale bar is 0.1 µm everywhere.

As a further proof of uranium accumulation in transgenic plant leaves, their ultrastructure was analyzed by transmission EM without any additional heavy atom staining. It was reasoned that uranium accumulated in the transgenic leaves makes them electron-dense compared to control leaves grown on uranium but having negligible uranium content. One line per construct was used for EM without any staining to detect uranium species in leaves of the plants grown on uranium-containing medium (i 4). There, pictures of the control root cells have black needle-shaped and flaky crystals. These are apparently made from uranyl ion complexes with organic acids secreted by cells [9] and these crystals are located at or near the plasma membranes (Figure 4a). Untransformed leaves had neither crystals of any kind, nor any dark areas between chloroplast membranes (Figure 4b). Leaves transformed by RbcS-c3 had electron-dense intermembrane space of chloroplasts around the starch grains (Figure 4f), and so did the plant leaves carrying RbcS-c1c3 (Figure 4c). Beyond that, RbcS-c1c3 cells formed uranium-containing structures in their chloroplasts that were obviously different from uranyl complexes on the root surface (see Figure 4a). The elemental analysis of these structures has not been done, but beside uranium the samples contained no heavy atoms that would be able to produce electron-dense structures following all the procedures for TEM samples preparation. Some elements (specks) that these structures are made of are as small as a few nanometers (Figure 4d inset). The structures were all well-above the micrograph background, see Figure S1.xlsx in Supplementary materials.

**Figure 5:**
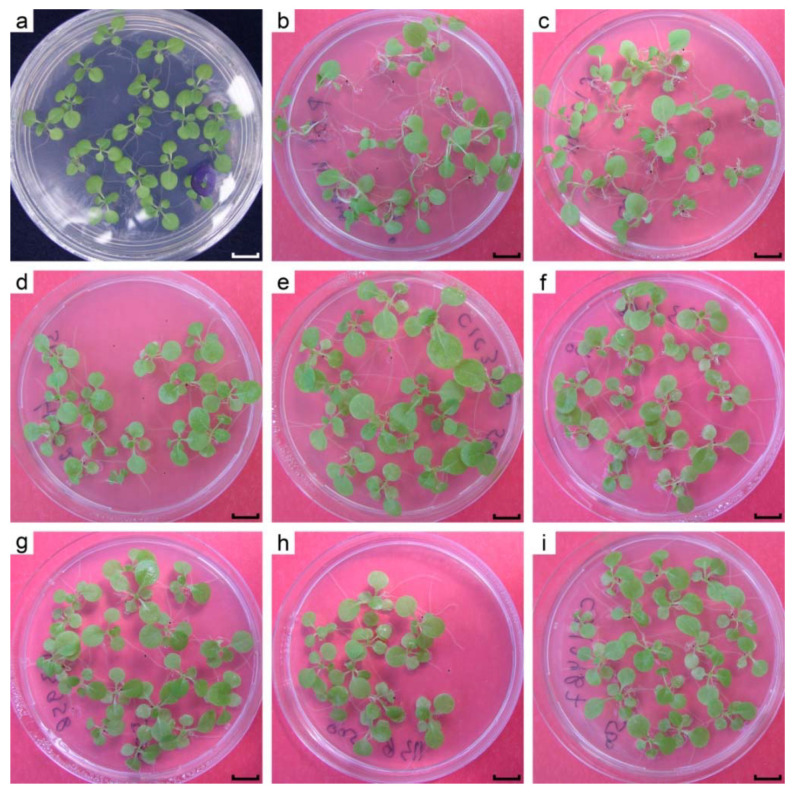
Tobacco seedling grown from seeds. All plates are 90 mm in diameter. (**a**) the control pBI121 line grown for 16 days on uranyl-free medium. (**b**–**i**) transgenic lines grown for 34 days with 500 µM uranyl: (**b**) the control pBI121 line; (**c**) c1c3-111; (**d**) c1c3-48; (**e**) c1c3-74; (**f**) c3-116; (**g**) c3-95; (**h**) c3-113; (**i**) c3-124. The lines are designated as construct name-line number. RbcS has been omitted in construct names for brevity.

**Figure 6:**
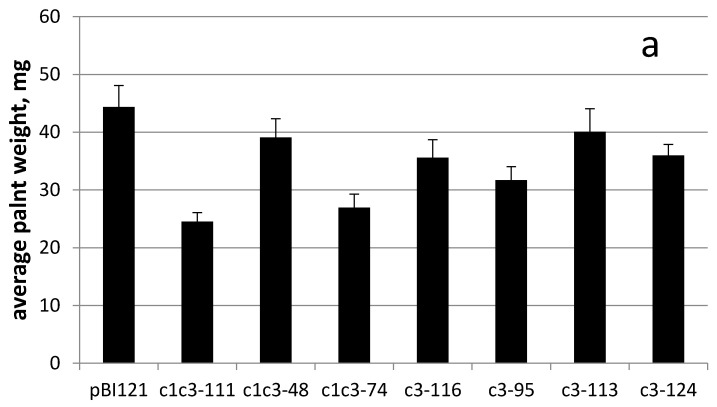

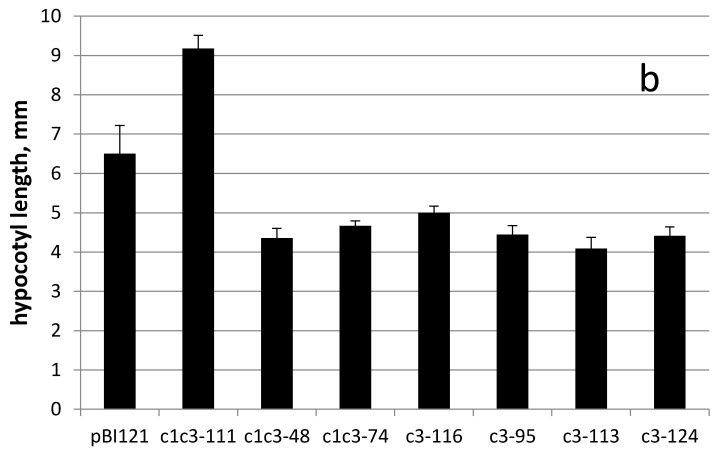
Fresh weights (**a**) and hypocotyl lengths (**b**) of tobacco seedlings from Figure 5.

Introduction of uranium reduction to bacteria was reported to be accompanied by an increase of its tolerance to uranium [27]. Henceforth, it was hypothesized that uranium reducing transgenic plants would be more tolerant to uranium compared to wild type plants. Firstly, the response of the plants to different levels of uranyl acetate in the growth medium was evaluated. Regardless of genotype, 800 µM uranyl acetate almost completely inhibited root growth at 16 days post germination, and the true leaves appeared, but did not grow larger than 3 mm, whilst all plants somehow were able to cope with as much as 600 µM of uranyl in the medium. Therefore, 500 µM uranyl acetate was chosen for comparison of control and cytc3-transformed plant lines. Plants were grown from seeds on MS medium supplemented with 500 µM uranyl acetate for 34 days and were then photographed, weighed and had their hypocotyls lengths measured (Figure 5 and Figure 6). Shown at Figure 5a are the phenotypically normal *N.tabacum* cv. Petit Havana grown for 16 days from seeds on uranium-free MS medium. When 500 µM of uranyl acetate was added to the medium, the control plants grew to approximately the same size for 34 days (Figure 5b), pointing to about twofold retardation of the seedling growth by 500 µM uranyl. Beside slowing the growth, uranyl toxicity led to developmental abnormalities of the root system, stems and cotyledonous and true leaves. The control plants grown on uranyl had long and curved roots and petioles. This stretching of plant organs had been reported by us [28] and elsewhere. The cotyledonous leaves of control plants were small and bent downward (see Figure 5b). On the other hand, most lines transformed by either cytc3 construct had rather normal phenotype (Figure 5c–i). Interestingly, all cytc3 transgenic lines grown for 34 days on 500 µM uranyl had smaller weights possibly because they were less stretched by uranyl in the medium (Figure 6a). This idea is further supported by hypocotyls measurements of control and cytc3-transgenic plants grown on uranyl. With exception of c1c3-111, all transgenic lines had shorter hypocotyls compared to control plants (Figure 6b). Also, the c1c3-111 line morphology (Figure 5c) is similar to that of control plants (Figure 5b). Cytc3-transgenic plants had less developed root systems. Their roots were mostly straight and somewhat shorter than those of control plants, indicating of the ability to grow faster with no negative effects of disproportionate growth. The transgenic plants also had shorter petioles and larger cotyledonous and true leaves (Figure 5c–i), they looked normally developed with no defects or abnormal bends. Their leaves were optimally situated so that the rosettes are open upward for better absorption of light for photosynthesis.

## 3. Discussion

Uranium (VI) can apparently enter bacterial cells of different species and accumulate there to toxic levels [27]. A paper by Huang et al. [6] also points to uranium entering plant cells, as without entering cells high accumulation of uranium on mere root surface would seem unlikely.

Most studies on uranium uptake by bacteria, for example [29] indicate that uranium is being reduced in periplasm. By now, reduction and therefore detoxification and accumulation of uranium has been demonstrated in a few bacteria species [12,30]. When correctly targeted, cytc3 was hypothesized by us to reduce uranium in plant leaves serving two purposes. First of all, it would create a gradient of uranyl ions to make uranyl flow to leaves, and second of all, it would convert uranyl to less toxic U(IV) species and thus increase plant tolerance to uranium. When a protein encoded by mitochondrial genome is expressed from a nuclear chromosome, it is said to be allotopically expressed [31]. A bacterial protein cytochrome c3 is targeted to periplasm in its native *D. vulgaris*. In our work, the cytc3 coding region was introduced in tobacco and fused, at its N-terminus, to the cleavable leader of potato cytochrome c reductase, with the latter being a nuclear coded protein targeted to IMS of mitochondria in potato. Another construct used here had no leader sequence at the N-terminus of cytc3 apoprotein, since cytochrome c3 size of 13,976 Da appears to be small enough to cross outer membranes of mitochondria [32] and possibly chloroplasts [33]. In either construct, cytc3 expression was driven by RbcS promoter specific for photosynthetic organs [24,26]. The purpose of using this promoter was to avoid cytc3 activity in roots, where it would be difficult to distinguish cytc3-reduced uranium inside root cells from uranium in the medium or on root surface (see Figure 5a). Also, uranium reduced in roots would be insoluble and not transported to the above-ground organs. The cytc3 gene was introduced in tobacco, a model species amenable to genetic transformation that quickly grows large biomass to easily measure uranium accumulation. Not surprisingly, cytc3 expression in different transgenic lines varied (Figure 2), and the lines with the highest expression proved to accumulate the highest concentrations of uranium ranging from 20 to 28 µg/g in their leaves (Figure 3, lines c1c3-74 and c3-124). Huang et al. [6] screened plant species and found a hyperaccumulator, *Brassica chinensis*, that takes up to 5 µg/g uranium in its above-ground fresh biomass. Thus, introduction of cytc3 to a model species may turn it to a uranium hyperaccumulator even without any special treatments of the growth substrate.

To further confirm uranium uptake by cytc3-transgenic plants, the leaves ultrastructure was analyzed by TEM. To detect uranium, the samples were prepared without any additional heavy atom staining, so the contrast of the photographs was low, but any electron-dense structures were to contain uranium. The ability of cytc3 to reduce uranium, the different appearance of these structures from crystals on the root surface and uranium hyperaccumulation by the transgenic leaves all point to those structures being nanoparticles of uraninite. The fact that uraninite crystals were detected in chloroplasts, not mitochondria, is not surprising, for some leaders were reported to mistarget proteins to a wrong cellular compartment [34]. No data are available on absence of such mistargeting by cytochrome c1 reductase leader peptide used in our studies. Besides, chloroplasts are much easier to visualize than mitochondria by TEM due to their abundance and larger sizes in leaf cells. Such particles of either biogenic or chemical origin have been reported (see [30] and the refs. within). The authors of [30] also present TEM photographs of the samples produced without heavy-atom staining.

In the experiments on uranium accumulation, the plants were grown on 200 µM uranyl that did not change tobacco growth rate or morphology (data not shown). Of our interest was a question whether cytc3-transgenic plants tolerate the toxic levels of uranium better than wild type plants do. Neither control nor transgenic lines grew on 800 µM of uranyl, but 500 µM uranyl caused growth defects of only the control plants (see Figure 5). In that experiment, the plants were grown from seeds germinated on agar medium supplemented with uranyl. The possible explanation for the transgenic plants not surviving the letal 800 µM of uranyl could be that the cytc3 gene was not expressed in them until the leaves opened and uranium was not reduced and detoxified yet then, at the seedlings stage. But in established seedlings, cytc3 expression alleviated uranium toxicity and the transgenic plants develop normally.

The perspectives of this work may involve trying ubiquitous or root-specific promoters to see if they may raise the lethal threshold concentration of uranyl. At the same time, cytc3 activity in leaves may make plants accumulate uranium in the above-ground organs at rates similar to described in this paper. A ubiquitous and strong promoter may help plants survive high uranyl, but the question of practical uses of such plants remains. Even at contaminated sites, uranium is seldom present at concentrations that are toxic to plants, yet uranium there would be still harmful to humans. Among other improvements one can think of further cytc3 gene modifications, transforming uranium hyperaccumulators *B.chinensis* or/and *Lemna* spp. and field applications at industrially or naturally uranium-contaminated sites like Sillamae (Estonia) or Zhovti Vody (Ukraine).

## 4. Materials and Methods

The mature cytochrome c3 coding region was amplified from *Desulfovibrio vulgaris* str. Hildenborough genomic DNA kindly provided by All-Russian Collection of Microorganisms at Skryabin Institute of Biochemistry and Physiology of Microorganisms, Pushchino, Russia. The PCR fragment was cloned in NotI and SacI sites of pBluescriptII KS(+) (Stratagene, La Jolla, CA, USA), excised from the resulting plasmid with XbaI and SacI and cloned in XbaI and SacI sites of pBI121 (Clontech) in lieu of GUS. RbcS promoter was amplified from DNA of *N. tabacum* DNA cv. Samsun. The cytochrome c1 reductase leader was reverse transcribed and amplified from RNA of *S. tuberosum* cv. Desiree kindly provided by A.S.Burgutin of K.A.Timiryazev Institute of Plant Physiology RAS. All PCR fragments were obtained using Taq DNA polymerase (Sibenzyme, Novosibirsk, Russia), cloned in pTZ57R/T (Fermentas, Vilnius, Lithuania) and sequenced. RbcS promoter and cytochrome c1 oxidase leader coding region restriction enzyme fragments were ligated in pBI121 modified as described above. The respective restriction enzymes and primers are given in RbcS-c1c3.dna and RbcS-c3.dna files in Supplementary materials. The construct assembly was done by standard methods [35]. The constructs were introduced in *A.tumefaciens* str. AGL1 [36] by a freeze-thaw procedure [37] and used to transform *N.tabacum* cv. Petit Havana as described [38]. The pot-grown transformants (T0) were selfed, and their progeny (T1) were selfed as well, and the T3 seeds from T2 plants checked to be Km-resistant by germinating on MS medium (Merck St. Louis, MO, USA) with 0.5% sucrose and 50 mg/L Km were used for further analyses. All plants were grown in a growth chamber at 22–24 °C under 10,000 Lux of white light on a 14/10 cycle. For DNA and RNA isolations, plants were grown from seeds in Magenta boxes on MS medium with 0.5% sucrose and 50 mg/L Km. RNA was isolated as described in [28]. For Northern hybridizations, RNA was blotted to Amersham Hybond-N+ membrane (GE Healthcare, NY, USA) and probed with XbaI-SacI fragment containing cytc3 coding region that was ^32^P-labelled by NEBlot kit (New England Biolabs, MA, USA). Blotting, hybridization and membrane washing was done according to instructions to Amersham Hybond-N+ membrane, and hybridization signals were detected using Typhoon 8600 PhosphoImager (Molecular dynamics, Chatsworth CA, USA) gel scanner and software. RT-PCR was done using M-MuLV reverse transcriptase, Taq polymerase (Sibenzyme, Russia) and the primers used for cloning cytc3 coding region. The PCR was carried out for 35 cycles consisting of 30 s denaturation at 92 °C, 30 s annealing at 60 °C and 1 min synthesis at 70 °C. For uranium accumulation, plants were grown from seeds in Magenta boxes on MS medium with 0.5% sucrose, 50mg/L Km and 200µM filter-sterilized uranyl acetate. Then the above-ground part was cut from roots above agar surface, incinerated at 600 °C for 3h and the ashes were dissolved in 10 mL 2% nitric acid. Uranium concentration in the samples was measured with Liberty Series II ICP-OES (Varian International Inc., Palo Alto CA, USA). For TEM, root tips and leaf discs from central parts of the third true leaves were fixed in 2.5% glutaraldehyde in 0.1 M Sorensen’s buffer pH 7.2 at 4 °C with 15 g/L sucrose for 2 days, followed by two rinses in the same buffer. The samples were then dehydrated by a series of 30 min incubations in 30%, 50%, 70%, 96% and 100% ethanol, transferred to ethanol:propylene oxide 1:1 mixture for 5 min and to pure propylene oxide. The samples were polymerized in Epoxy embedding medium (Merck) for 2 days at 56 °C. Using Ultrotome V (LKB, Bromma, Sweden), ultrathin sections were prepared and placed on Formvar-coated copper grids. The samples were viewed and photographed at an X-500 electron microscope (Hitachi, Japan). To assay seedlings response to uranyl, tobacco seeds were sterilized for 5 min in 0.5% sodium hypochlorite, washed twice by sterile water and placed on MS medium with 0.5% sucrose, 50 mg/L kanamycin sulfate and 500 µM uranyl acetate in Petri plates. The plates were kept at 4 °C in dark for 3 days and moved to growth chamber for 34 days.

## Data Availability

Data are contained within the manuscript.

## Supplementary Materials

The following are available online at https://www.mdpi.com/article/10.3390/ijms222312622/s1.
