# Supplementary figures and images for: Expression of Cytochrome c3 from Desulfovibrio vulgaris in Plant Leaves Enhances Uranium Uptake and Tolerance of Tobacco
Source: Int J Mol Sci. 2021 Nov 23;22(23):12622. doi: 10.3390/ijms222312622 (PMC8657950; doi:10.3390/ijms222312622)

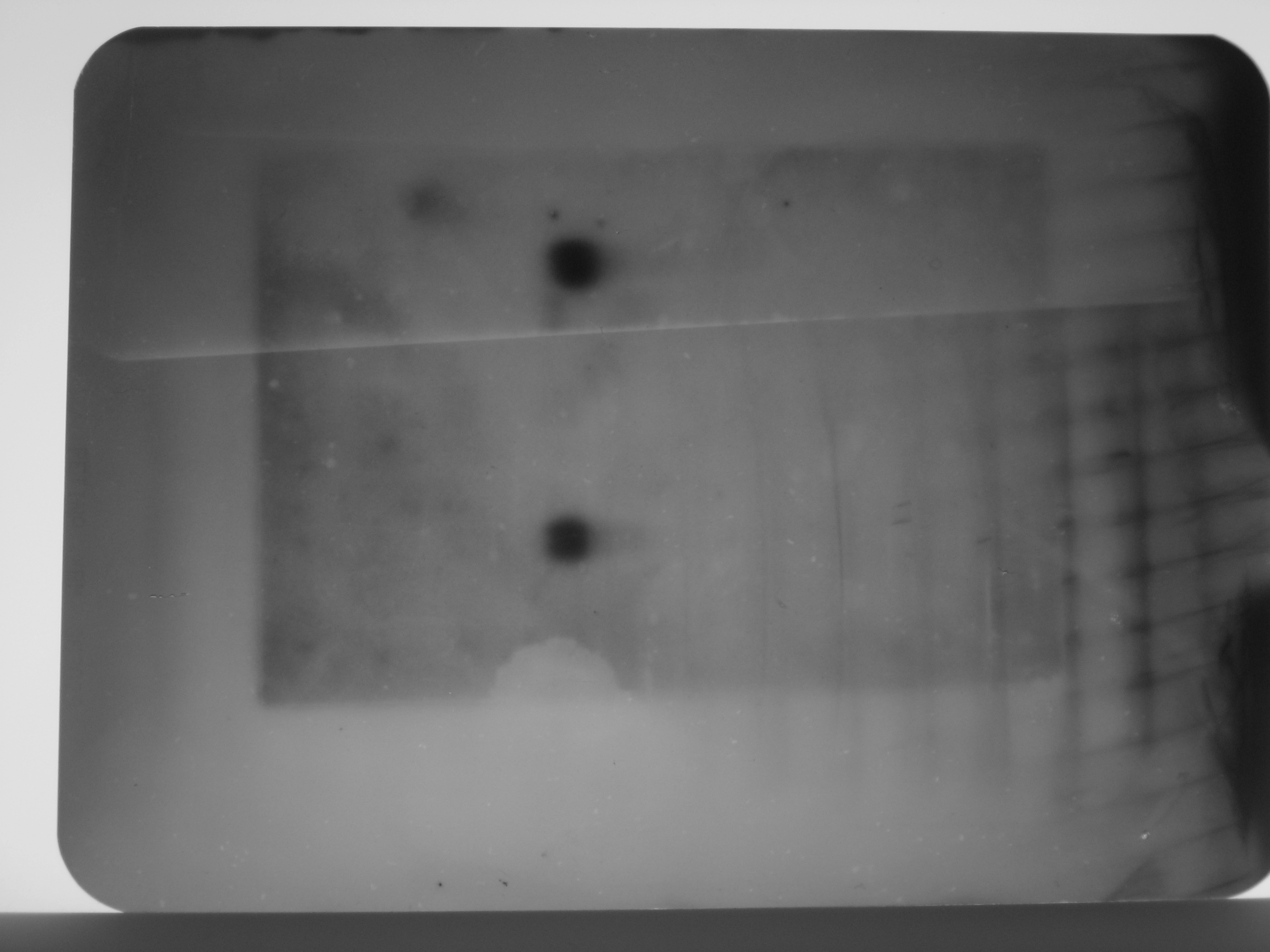

Supplement: Supplementary file 1 [file ijms-22-12622-s001.zip › rnac3.jpg]
